# Light color and the commercial broiler: effect on behavior, fear, and stress

**DOI:** 10.1016/j.psj.2022.102052

**Published:** 2022-07-13

**Authors:** B. Remonato Franco, T. Shynkaruk, T. Crowe, B. Fancher, N. French, S. Gillingham, K. Schwean-Lardner

**Affiliations:** ⁎ Department of Animal and Poultry Science, University of Saskatchewan, Saskatoon, SK, Canada, S7N 5A8; † Department of Mechanical Engineering, University of Saskatchewan, Saskatoon, SK, Canada, S7N 5A9; ‡ AviagenTM, Huntsville, AL 35806, USA

**Keywords:** light color, broiler, behavior, fear, stress

## Abstract

Light is an important component in poultry production, and it may impact bird behavior, an important component of animal welfare. Light-emitting diode (**LED**) lamps are of interest for broiler production since they are inexpensive to run and provide monochromatic colors. This study aimed to understand the impact of three light colors (blue, green, or white), provided by LED lighting, on behavioral expression, stress and fear levels of broilers. A total of 14,256 male and female broilers of 2 genotypes (Ross EPMx708 and Ross YPMx708) were housed in 9 rooms in 2 blocked trials (3 room replicates per light per trial), with sexes and genotypes housed in 12 separate pens per room. Behavioral expression was recorded using an infrared camera and analyzed using a scan sampling technique. To assess fear, 3 tests were conducted: tonic immobility, novel object, and response to observer. Blood was collected to evaluate chronic stress using the heterophil:lymphocyte (**H:L**) ratio. Data were statistically analyzed using SAS (MIXED procedure) in a 3 × 2 × 2 factorial design, with lighting treatment nested within room. Fear tests indicated reduced fear levels in birds raised under blue light (lower latency to rise during the tonic immobility test and a lower percentage of birds moving due to the passage by of an observer). No differences were observed for the novel object test. Light color resulted in changes in stress levels, indicated by a lower H:L ratio for broilers raised under blue light compared to those raised under white light. Behavior was influenced by light color, especially at 33 to 34 d of age, where birds raised under white light were more active, and birds raised under blue light spent more time resting. Overall, results indicated that light color has minor influences on behavioral expression. Utilizing blue light during the brooding and rearing phase leads to lower stress and a reduction in fear, suggesting that blue light may improve the emotional states of fear and stress, thereby improving the welfare of poultry.

## INTRODUCTION

In a light-tight, environmentally controlled poultry house, artificial light is provided to broilers during the photophase. Since light can have significant impacts on production, physiology, health, and behavior, the choice of lighting system is important (Prayitno et al., 1997a; Alvino et al., 2009; Deep et al., 2012; Schwean-Lardner et al., 2012). Light programs in poultry barns vary by manipulating various components, including photoperiod, intensity, and wavelength (light color).

There is increasing attention in literature with respect to spectrum as part of broiler lighting programs, specifically as it relates to the movement toward light-emitting diode lighting (**LED**). These lights have several advantages over other light sources, such as a longer life span, small size, low thermal output, and adjustable light intensity (Yang et al., 2016). In addition, LED light bulbs also have the potential to provide specific monochromatic color.

Poultry species possess a highly specialized visual system, and although they share many characteristics with mammals, birds possess superior vision due to several physiological adaptations. Birds are tetrachromatic and have a peak of sensitivity similar to humans, between wavelengths of 545 to 575 nm (green light). However, birds possess 2 other peaks of sensitivity which humans do not. Those peaks occur at wavelengths of approximately 400 to 480 nm (blue) and 580 to 700 nm (orange/red) (Lewis and Morris, 2000). Birds also have a double-cone photoreceptor, which is thought to mediate achromatic motion perception (Yang et al., 2016). In addition, birds possess oil droplets in their cone cells, increasing color vision and allowing them to perceive UV-light (Lewis and Morris, 2000). Because of those characteristics, birds likely perceive color differently, with some colors appearing extra bright for birds compared to humans' sensation. This fact should be considered when developing a lighting program. The typical measurement of light intensity, reported in lux or foot-candles, is based on human spectral sensitivity. A measure that considers bird spectral sensitivity, called clux, or chicken/corrected lux, can be used and gives a more reliable indication of how birds perceive light (Prescott and Wathes, 1999, Prescott et al., 2003).

Ideally, light provided in poultry houses should allow birds to perform critical tasks and should not compromise the physiological process of vision (Prescott and Wathes, 1999). The light program chosen can alter birds’ ability to perceive their environment, which may alter their ability to perform normal behaviors. For example, an environment that only provides red light may affect a hen's ability for social recognition since the pale appearance of combs makes distinguishing specific hens more difficult (D'Eath and Stone, 1999). More research is needed on the impact of light wavelength on the recognition of objects, navigation around the poultry house and fear levels (Prescott et al., 2003).

In addition to vision, light is responsible for the regulation of other processes. Behavior, for example, can be mediated by other non-visual photoreceptors (Prescott and Wathes, 1999), leading to non–image-forming or non-visual responses. In this situation, light can be received directly by the pineal gland, which is responsible for synthesizing serotonin and melatonin (Archer, 2019). Other deep brain, or encephalic, photoreceptors, can also receive light. These are primarily present in hypothalamic regions of the brain (Kuenzel et al., 2014), and play a role in regulating diurnal rhythms (Lewis and Morris, 2000). The ability to stimulate non-visual photoreceptors is partially dependent on light wavelength, and there is an interaction between wavelength and light intensity. Low wavelength light requires higher intensities to pass through the skull and activate non-visual photoreceptors (Lewis and Morris, 2000; Baxter et al., 2014). In humans, light wavelength can also generate responses by activating a non-classical photoreception mediated by the intrinsically photosensitive retinal ganglion cells system. This system is modulated by the photopigment melanopsin, which is most efficiently excited by short wavelengths and can change behavior, alertness, and cognition (Vandewalle et al., 2010). In birds, horizontal cells appear to exert a dual function, regulating visual and non-visual tasks by expressing melanopsin (Morera et al., 2016).

Previous research has indicated that wavelength treatments can affect the behavior of chickens. Prayitno et al. (1997a,b) has reported that broilers raised under red and white light were more active, whereas birds raised under blue and green light spent a higher percentage of time sitting and dozing. Hesham et al. (2018) demonstrated that broilers raised under red light displayed higher levels of aggression and feather pecking due to increased activity. Broilers exhibited less fear (Mohamed et al., 2017) and aggressive behavior, were less active (Khaliq et al., 2018) and displayed more comfort and nutritive behaviors when raised under short wavelengths, such as blue and green (Lucena et al., 2020). The authors believed this effect was due to photons of longer wavelength being more effective at penetrating the hypothalamic photoreceptors than those of shorter wavelength, a characteristic that was demonstrated by Hartwigg and van Veen (1979). However, comparing different wavelength trials and their results on behavior can be complicated since many have not equated illuminance to broiler spectral sensitivity (Lewis and Morris, 2000). Therefore, there may be cofounding results of light wavelength and intensity.

The present study investigated the effects of various wavelength treatments (resulting in blue, green, or white light) on broiler chicken behavior, fear, and stress levels when light intensity was based on avian spectral sensitivity.

## MATERIALS AND METHODS

This experiment was approved by the Animal Care Committee of the University of Saskatchewan. It was conducted following the guidelines of the Canadian Council on Animal Care (2009) as specified in the Guide of the Care and Use of Experimental Animals.

### Housing and Management

This data set was part of a more extensive study, focusing on the impact of wavelength treatments, genotype and sex on broiler production and welfare. Broilers were raised from 0 to 35 d in one experiment with 2 blocked trials. The trials were designed to mimic commercial production practices, with a total of 7,128 feather-sexed broilers housed for each trial. The research facility contained 9 individually controlled rooms. Each room was subdivided into 12 pens (2 × 2.3 m). Males and females as well as genotypes (Ross YPMx708 and Ross EPMx708, males and females) were housed in separate pens within rooms, allowing for a total of 6 room replications per lighting treatment and 18 replicate pens × sex × genotype × lighting program over the 2 trials.

The estimated final stocking density was 31 kg/m^2^ (62 males or 70 females per pen). Litter consisted of an equal amount of wheat straw in each pen (approximately 7.5–10 cm depth). In each pen, one aluminum tube feeder (110 cm of pan circumference from 0 to 30 d and 137.5 cm from 30 d to market) and one drinker line (6 pendulum nipple drinkers) provided birds' water and feed ad libitum. Supplemental feeders and drinkers were provided during wk 1. Commercially produced diets were formulated based on Aviagen's Ross 708 requirements, with a starter diet amount of 0.5 kg of feed/bird, grower diet of 2 kg/bird and the balance fed as finisher diet until 35 d.

The temperature was monitored daily in each room and assessed via computer reading and observation of bird behavior. Heat was provided through wall-mounted hot water pipes in each room. Initial temperature was set at 32.1°C on d 0 and was gradually reduced until reaching 21°C by d 25, where it was maintained until d 35. During the early brooding period, humidifiers were placed in rooms at d 0 and removed at d 5 to ensure 40 to 60% relative humidity.

### Lighting

**Figure 1:**
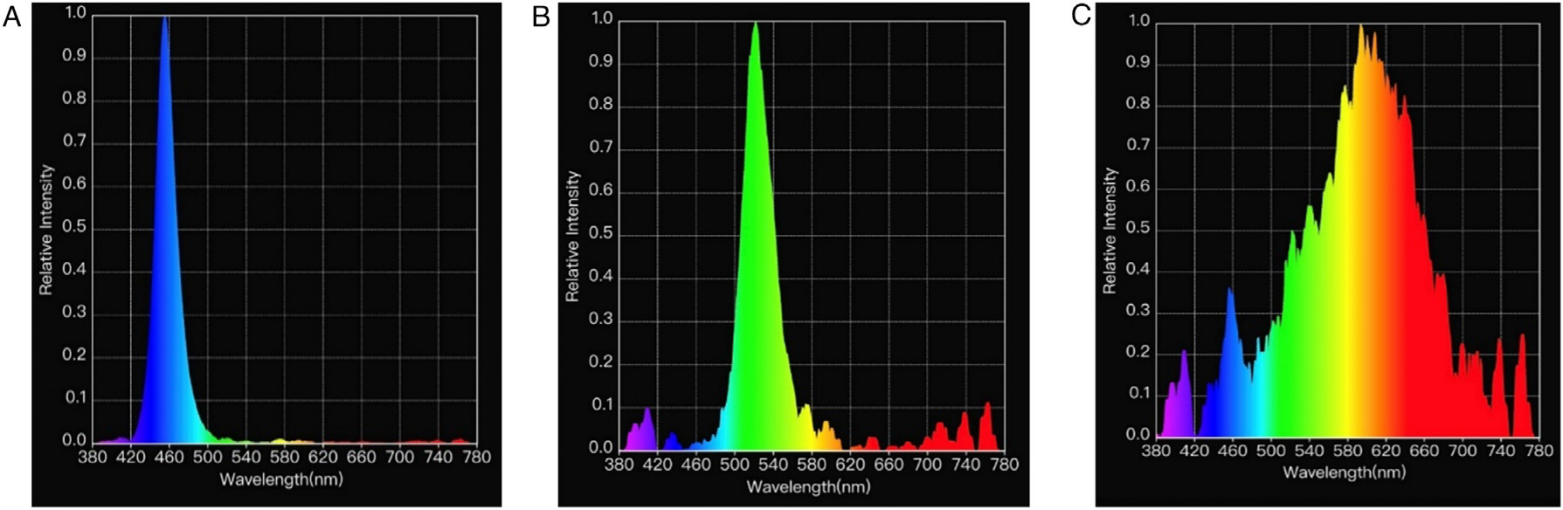
Measurements of light spectrum respectively from the blue (A, peak at 455 nm), green (B, peak at 510 nm), and white (C) treatments.

In each room, LED light bulbs (11W Alice Non-Directional LED Lamps, Greengage Agritech Limited, Roslin Innovation Centre, University of Edinburgh, Easter Bush Campus, Midlothian, EH25 9RG, United Kingdom) provided lighting for specific light treatments (3 rooms per lighting wavelength treatment or color). Colors corresponded to blue (dominant wavelengths near 455 nm), green (dominant wavelengths near 510 nm), and white (combination of wavelengths). Light spectrum was confirmed using the light meter Lighting Passport (Asensetek Incorporation, New Taipei City, Taiwan). The measurements of the spectrum of light from each experimental treatment are shown in Figure 1.

Photoperiod length was 23L:1D on d 0. Daylength was reduced gradually (1 h per day) until reaching 18L:6D at d 5. Dawn and dusk periods of 15 min were provided daily before lights were fully on and off (time included in the photophase). Light intensity, measured at bird head height, was set at specific clux levels using a Hato Galilux Light Meter (based on bird spectral sensitivity (Lewis and Morris, 2000). For trial 1, light intensity was 9.6 ± 0.4 clux for the entire trial. Within trial 2, the intensity for the first week was set for 14.3 ± 0.1 clux and the remaining weeks 9.6 ± 0.4 clux.

### Data collection

A summary of data collected, time of collection, and number of birds or pens per light treatment × genotype × sex per trial are presented in Table 1

**Table 1:** Summary of data collected, time of collection and number of pens or birds per light × genotype × sex per trial (2 trials).

Data	Time of collection	Number of pens or birds per light × genotype × sex (per trial)
Behavioral observation	11–12 days and 33–34 days	2 pens
Tonic immobility	23 days	12 birds
Novel object test	30 days	3 pens
Response to observer test	30 days	9 pens
H:L ratio	22 days	9 birds

#### Behavioral Observations

Bird behavior was assessed at 2 ages within each trial (11–12 and 33–34 d). Infrared cameras (Panasonic WV-CF224FX; Panasonic Corporation of North America, One Panasonic Way 7D-4, Secaucus, NJ) were mounted on ceilings above pens so that the entire area where animals were housed was captured. Behavioral expression was recorded for a 24-h period at each of the 2 ages, in one pen from each combination of genotype × sex within 2 rooms per lighting treatment (2 pens per light × genotype × sex for 24 pens total per trial). Scan sampling was conducted at 20 min intervals, assessing the behaviors described in the ethogram provided in Table 2 (Schwean-Lardner et al., 2012).

**Table 2:** Ethogram (Schwean-Lardner et al., 2012).

Behavior	Description
Feeding	Bird at the feeder, with the head into the lip of the feeder
Drinking	Bird standing with the head directly under the drinker line
Inactive resting	Bird lying on the straw and not performing any other behavior – may or may not be sleeping
Walking	Bird taking more than two consecutive steps
Standing	Bird in an upright position with both feet on the ground (but no other part), idle (not performing any other behavior)
Running	Bird running for more two seconds or more
Preening	Bird manipulating feathers on own body. May be lying or standing
Leg or wing stretching	Bird stretching leg or wings to the side or behind the body, without taking a step forward, or flapping
Dustbathing	Bird in a sitting position, shaking wings vertically, followed by side or head rubs, involving the motion of the legs
Foraging	Manipulating litter, feed with beak, previous or after scratching substrate with feet

#### Fear Tests

To assess the impact of treatment on bird fear response, 3 tests were performed:

• *Novel object test* (Forkman et al., 2007)*:* In this test, the observer placed a novel object (bright and colored) slowly in the center of each pen, then retreated outside of the pen. The observer then timed how long it took for the first 3 birds to peck at the object. Four pens per room were assessed (one pen per sex × genotype; total of 3 pens per light × genotype × sex × trial) at 30 d of age in each trial.
• *Response to observer test* (Schwean-Lardner et al., 2012)*:* For this test, an observer walked slowly past each pen while recording the behavior of the birds using a handheld video camera (Canon Vixia HFR700 camcorder; Canon Canada, Mississauga, ON, Canada). Videos were then analyzed by counting how many birds moved as a result of the passage of the observer, and results were reported as percentages. All pens were analyzed at 30 d of age, resulting in measures from 9 pens per light × genotype × sex per trial,
• *Tonic immobility test* (Jones and Faure, 1981): Birds were placed on their backs in a U-shaped saddle and manually restrained for 15 s. Latency to rise was measured in seconds. If birds stayed in the tonic state for more than 600 s, they were removed from the saddle, and their tonic immobility duration was quantified at 600 s. Birds were assessed at 23 d of age (12 birds per lighting treatment × genotype × sex, 3 rooms per lighting treatment).

#### Stress

To assess birds’ stress levels, heterophil: lymphocyte (**H:L**) ratio was calculated (Gross and Siegel, 1983). Blood samples were collected from broilers at 22 d from the brachial vein into a 2.0 mL vacutainer tube containing EDTA. In each trial, samples were obtained from one bird per pen per room, or 9 birds per light × genotype × sex (total of 108 samples in each of the 2 trials). Tubes were placed on a mixer for 5 min following collection prior to creating blood smears on slides, then later stained using PROTOCOL Hema 3 (Fisher Scientific, Ottawa, Canada). To determine the H:L ratios, slides were read using a field of view of 100X oil magnification until 100 cells were counted.

### Statistical Analyses

Data were statistically analyzed using SAS (SAS 9.4, Cary, NC). Data were verified for normality using PROC UNIVARIATE and log-transformed if needed prior to analyses. Data was then tested as a 3 × 2 × 2 factorial design with lighting treatment nested within room (MIXED procedure). Replicate unit was room for wavelength treatment and pen for sex and genotype. Tukey's test was used to determine mean separation. Differences were considered significant when *P* ≤ 0.05.

## RESULTS

### Behavioral Observations

The behavioral expression results are shown in Table 3 (11–12 d) and 4 (33–34 d). At 11 to 12 d, light color had an impact on preening behavior only, where broilers raised under blue light preened for a higher percentage of time than birds raised under green light (blue: 0.88%, green: 0.54%, white: 0.77%, *P* = 0.05). At this age, females spent an increased percentage of time drinking (females: 5.59%, males: 4.63%, *P* = 0.04) and dustbathing (females: 0.24%, males: 0.14%, *P* = 0.002) compared to males, but males spent a larger percentage of time displaying foraging behavior (males: 1.58%, females: 1.20% *P* = 0.05).

**Table 3:** Effect of wavelength treatment^1^, genotype, and sex, on broiler behavioral observations as a percentage of time over 24-h time periods at 11–12 days of age.

	Light				Genotype			Sex			
	Blue	Green	White	*P*-value	Y-708	E-708	*P*-value	Male	Female	*P*-value	SEM^2^
Nutritive											
Feeding	9.75	9.89	10.58	0.51	9.78	10.37	0.27	10.38	9.77	0.38	0.017
Drinking	4.95	5.37	5.18	0.63	5.02	5.30	0.48	4.63^b^	5.59^a^	0.04	0.022
Mobility											
Standing	4.91	4.18	4.39	0.48	4.52	4.47	0.91	4.72	4.26	0.29	0.028
Walking	4.92	5.12	5.17	0.69	4.96	5.18	0.70	5.22	4.92	0.06	0.018
Running	0.81	0.75	0.91	0.30	0.76	0.88	0.90	0.82	0.83	0.67	0.018
Inactive/ resting	72.02	72.11	71.30	0.74	72.43	71.19	0.10	71.22	72.41	0.10	0.006
Other behaviors											
Preening	0.88^a^	0.54^b^	0.77^ab^	0.05	0.70	0.76	0.54	0.73	0.73	0.79	0.028
Leg/wing stretch	0.26	0.28	0.21	0.36	0.27	0.23	0.26	0.26	0.24	0.67	0.013
Dustbathing	0.20	0.21	0.16	0.89	0.21	0.18	0.44	0.14^b^	0.24^a^	0.002	0.010
Foraging	1.32	1.55	1.31	0.60	1.34	1.44	0.57	1.58^a^	1.20^b^	0.05	0.028

1 Specific wavelength treatments were blue (dominant wavelengths of 435-500 nm), green (dominant wavelengths of 500-565 nm) and a combination of wavelengths to produce white light.

2 SEM = Standard error of the mean.

a,b Means with common letters do not differ significantly (*P* ≤ 0.05)**.** No significant interactions were found between light, genotype, and sex.

Light color had a larger impact on behavioral expression at 33 to 34 d (Table 4). Birds raised under white light spent a higher percentage of time walking compared to those reared under other wavelength treatments (white: 8.15%, green: 3.41%, blue: 1.29%, *P* < 0.0001). Birds raised under blue light spent a higher percentage of time inactive/resting than birds raised under green or white light (blue: 79.96%, green: 75.65%, white: 72.81%, *P* = 0.03). YPMx708 broilers spent a higher percentage of time preening (YPMx708: 1.92%, EPMx708: 1.58%, *P* = 0.05) and leg/wing stretching (YPMx708: 0.79%, EPMx708: 0.30%, *P* = 0.002) than EPMx708 broilers. No significant differences between treatments were found in the percentage of time spent performing standing behavior.

**Table 4:** Effect of wavelength treatment^1^, genotype and sex, and significant interactions, on broiler behavioral observations as a percentage of time over 24-h time periods at 33–34 days of age.

	Light				Genotype			Sex			Interactions			
	Blue	Green	White	*P*-value	Y-708	E-708	*P*-value	Male	Female	*P*-value	Light × genotype	Genotype × sex	Light × genotype × sex	SEM^2^
Nutritive														
Feeding	4.91	7.20	5.83	0.005	5.99	5.97	0.82	6.11	5.85	0.57	-	-	0.01	0.022
Drinking	6.42	7.07	5.85	0.44	5.80	7.09	0.0001	6.23	6.66	0.07	-	-	0.008	0.039
Mobility														
Standing	3.50	3.41	3.67	0.26	3.29	3.70	0.14	3.46	3.53	0.26	-	-	-	0.037
Walking*	1.29^c^	3.41^b^	8.15^a^	<0.0001	4.73	3.90	0.23	4.60	3.97	0.49	-	-	-	0.119
Running	0.14	0.16	0.23	0.0007	0.14	0.21	0.003	0.16	0.20	0.06	-	-	0.009	0.009
Inactive/ resting	79.96^a^	75.65^b^	72.81^b^	0.03	75.97	76.31	0.24	75.93	76.34	0.33	-	-	-	0.009
Other behaviors														
Preening	1.79	1.70	1.76	0.54	1.92^a^	1.58^b^	0.05	1.66	1.84	0.10	-	-	-	0.024
Leg/wing stretch	0.52	0.53	0.58	0.94	0.79^a^	0.30^b^	0.0002	0.57	0.51	0.83	-	-	-	0.022
Dustbathing	0.22	0.18	0.32	<0.0001	0.27	0.21	0.02	0.29	0.19	0.0006	-	-	0.001	0.019
Foraging*	1.22^a^	0.74^b^	0.84^b^	0.002	1.1^a^	0.70^b^	<0.0001	0.93	0.86	0.74	0.01	0.01	-	0.027

1 Specific wavelength treatments were blue (dominant wavelengths of 435–500 nm), green (dominant wavelengths of 500-565 nm) and a combination of wavelengths to produce white light.

2 SEM, Standard error of the mean.

a,b Means with common letters do not differ significantly (*P* ≤ 0.05).

⁎ Data was log transformed to achieve normality.

In several cases, interactions were noted between wavelength treatment and behavior (Table 5). With respect to nutritive behaviors, an interaction was noted between light, ***genotype***, and sex, where EPMx708 females raised under green light spent a higher percentage of time at the feeder than did broilers from any other treatments (*P* = 0.01). For drinking behavior, EPMx708 females raised under white light spent the most time at the drinker (*P* = 0.008). For running behavior, EPMx708 males raised under white light, and EPMx708 females raised under green lights exhibited this behavior most (*P* = 0.009). For dustbathing behavior, YPMx708 males raised under blue light, YPMx708 females and EPMx708 males raised under white light spent a higher percentage of time dustbathing compared to the remaining treatments (*P* = 0.001).

**Table 5:** Behavioral observation interactions as a percentage of time over 24-h period at 33–34 days of age.

Interactions between light x genotype												
	Blue – Y-708	Blue – E-708	Green – Y-708	Green – E-708	White – Y-708	White – E-708						
Foraging	1.35^a^	0.90^ab^	1.07^a^	0.41^b^	0.88^ab^	0.79^ab^						

Interactions between genotype × sex												
	Y-708 Male	Y-708 Female	E-708 Male	E-708 Female								

Foraging	1.03^a^	1.17^a^	0.84^ab^	0.56^b^								


Interactions between light × genotype × sex												
	Blue Y-708 male	Green Y-708 male	White Y-708 male	Blue Y-708 female	Green Y-708 Female	White Y-708 Female	Blue E-708 male	Green E-708 male	White E-708 male	Blue E-708 female	Green E-708 female	White E-708 female
Feeding	5.12^de^	6.81^b^	6.83^b^	5.09^de^	6.51^bc^	5.55^d^	4.98^de^	7.29^ab^	5.63cde	4.43^e^	8.19^a^	5.32^de^
Drinking	5.48^ab^	6.04^ab^	5.62^ab^	6.14^ab^	6.60^ab^	4.94^b^	7.24^ab^	7.67^ab^	5.34^ab^	7.24^ab^	7.52^ab^	7.95^a^
Running	0.14^bc^	0.07^c^	0.13^bc^	0.21^ab^	0.13^bc^	0.14^bc^	0.10^bc^	0.10^bc^	0.39^a^	0.09^bc^	0.34^a^	0.26^ab^
Dustbathing	0.34^a^	0.17^bc^	0.30^ab^	0.18^bc^	0.26abc	0.36^a^	0.25abc	0.29abc	0.37^a^	0.10^cd^	0.001^d^	0.25abc

a,b,c,d,e Means with common letters do not differ significantly (*P* ≤ 0.05).

Interactions between light and ***genotype*** were found for foraging behavior, where YPMx708 broilers raised under blue and green light spent a larger percentage of time foraging than EPMx708 broilers raised under green light (*P* = 0.01). Interactions for this behavior were also noted between ***genotype*** and sex, where YPMx708 males and females displayed more foraging behavior compared to EPMx708 females (*P* = 0.01).

### Fear Tests

Results from the fear tests conducted (tonic immobility, response to observer, and novel object) are presented in Table 6. Birds raised under blue light had a shorter latency to rise in the tonic immobility test (blue: 71.79 s, green: 140.11 s, white: 150.41 s, *P* = 0.001). No differences between genotype or sex were found. In addition, a smaller percentage of birds moved due to the passage by of an observer compared to birds raised under green and white light (blue: 3.31%, green: 6.89%, white: 6.22%, *P* = 0.01). There was an interaction between genotype and sex, where a higher percentage of YPMx708 males moved compared to YPMx708 females in the response to observer test (*P* = 0.05, Table 6). The novel object test did not reveal any differences between birds raised under different light color, genotype, or sex.

**Table 6:** The effect of wavelength treatment^1^, genotype and sex on the tonic immobility test at 23 d of age, response to observer and novel object tests of broilers at 30 d of age.

	Light				Genotype			Sex			Interaction	
	Blue	Green	White	*P*-value	Y-708	E-708	*P*-value	Male	Female	*P*-value	Genotype × sex	SEM^2^
Tonic immobility												
Latency to rise (sec)*	71.79^b^	140.11^a^	150.41^a^	0.001	128.93	112.61	0.38	104.39	137.14	0.08	-	9.599
Response to observer												
% of birds moving from observer	3.31^b^	6.89^a^	6.22^a^	0.01	5.21	5.74	0.38	6.00	4.95	0.08	0.05	0.439
Novel object test												
Average time to approach novel object (sec)	105.8	131.3	163.5	0.12	149.5	117.1	0.16	139.9	126.7	0.56	-	13.284
Interactions between genotype × sex on the response to observer test												

	Y-708 male	Y-708 female	E-708 male	E-708 female								
% of birds moving from observer	6.34^a^	4.07^b^	5.66^ab^	5.83^ab^								

1 Specific wavelength treatments were blue (dominant wavelengths of 435–500 nm), green (dominant wavelengths of 500–565 nm), and a combination of wavelengths to produce white light.

2 SEM, Standard error of the mean.

a,b Means with common letters do not differ significantly (*P* ≤ 0.05).

⁎ Data was log transformed to achieve normality.

### Stress

Stress level results, assessed by examination of H:L ratio are presented in Table 7. Birds raised under blue light demonstrated a lower H:L ratio than those raised under white light (blue: 0.312, *P* = 0.03). Birds reared under green light were intermediate, with similar H:L ratios as those reared under blue and white light (green: 0.433, white: 0.467). With respect to sex, males had a lower H:L ratio compared to females (males: 0.370, females: 0.439, *P* = 0.001). No genotype differences nor interactions between treatments were noted.

**Table 7:** The effect of wavelength treatment^1^, genotype and sex on heterophil:lymphocyte (H:L) ratio of broilers at 22 d of age.

	Light				Genotype			Sex			
	Blue	Green	White	*P*-value	E-708	Y-708	*P*-value	Male	Female	*P*-value	SEM^2^
H:L ratio	0.312^b^	0.433^ab^	0.467^a^	0.03	0.399	0.409	0.63	0.370^b^	0.439^a^	0.001	0.0126

1 Specific wavelength treatments were blue (dominant wavelengths of 435-500 nm), green (dominant wavelengths of 500-565 nm), and a combination of wavelengths to produce white light.

2 SEM, Standard error of the mean.

a,b Means with common letters do not differ significantly (*P* ≤ 0.05).

## DISCUSSION

Behavioral expression is an important aspect of animal welfare. It is linked to positive or negative subjective states, such as comfort, empathy, fear, suffering, or pain. Environment characteristics, including lighting, may affect subjective states and the expression of behavior in broilers. While previous research studied the impact of wavelength treatments on various behavioral aspects, most have measured the intensity of light across various wavelengths (colors) in lux, which is based on human interpretation of light intensity. Under various light colors, this may have influenced the results obtained, as there was likely an interaction between wavelength and light intensity. Results from our study provide data where corrected lux (clux) was used to ensure intensity was similar across the various wavelength treatments.

Previous studies have provided inconsistent data with respect to the effect of wavelength treatments on nutritive behaviors. Results range from no impact of light color on nutritive behaviors (Sultana et al., 2020) to increased feeding behavior when broilers are raised under green or red lights (Sultana et al., 2013) or blue light (Hesham et al., 2018). In our study, in addition to short wavelengths, white light, which includes short and long wavelengths in its spectrum, also appears to influence nutritive behaviors. EPMx708 females raised under green light spent more time feeding, and when reared under white light these broilers spent more time drinking. These results may be correlated to the increased mobility behaviors expressed by birds raised under green and white light, as birds raised under blue light spent more time inactive.

Light color impacted the percentage of time birds spent performing mobility and resting behaviors. When raised under blue light, broilers spent a higher percentage of time inactive/resting. In contrast, white light influenced broiler mobility, where birds raised under white light exhibited walking behavior for a higher percentage of time. EPMx708 males raised under white light and EPMx708 females raised under green light spent a higher percentage of time performing running behavior. This agrees with previous research, which found that broilers raised under blue light were more inactive and spend more time sitting, while those raised under white light spent more time walking (Prayitno et al., 1997a, Sultana et al., 2013, Hesham et al., 2018).

In our study, comfort behaviors were displayed at low incidences, so care should be taken in interpretation of these data. Preening and leg/wing stretching were not affected by wavelength treatments. Preening is a maintenance behavior performed by birds to maintain healthy plumage (Alvino et al., 2009), and as with leg and wing stretching, performance of these behaviors may be important to animal wellbeing. Dustbathing behavior, another comfort behavior, was also only performed for a small percentage of time by broilers in all treatments: 1.22% of time for birds raised under blue light, 0.74% for birds raised under green light and 0.84% for birds raised under white light. For this behavior, an interaction was noted between light color, genotype, and sex, with an increasing percentage of time spent performing dustbathing by YPMx708 males raised under blue light and YPMx708 females and EPMx708 males raised under white light.

Fraser (2008) stated that assessing welfare should consider natural living, basic health and functioning and affective states, which consist of both positive and negative states. Fear, indicative of a negative affective state, is triggered by any perceived danger. In commercial flocks, several factors can elicit fear, including novelties in their environment and management, specific stimuli or social isolation (Forkman et al., 2007). Fear responses can range from avoidance, lack of approach, panic, piling, fighting, or immobility (Jones et al., 2015). In our study, the results of the fear tests indicated that birds raised under blue light had lower fear responses than birds raised under green and white light. This was indicated by a lower latency to rise during the tonic immobility test, and by a smaller percentage of birds moving as a result of the passage by of an observer. Previous studies have also demonstrated that raising broilers under blue light leads to a positive impact on fear levels, demonstrated through various fear tests, including tonic immobility (Sultana et al., 2013, Mohamed et al., 2014, 2017) and open field tests (Mohamed et al., 2017). Research has indicated that blue light exerts a calming effect on broilers (Prayitno et al., 1997a) and therefore, it has been cited as a positive tool to reduce fear during brooding and rearing, in routine management practices that may elicit fear, such as bird handling and other possible environmental stressors, or during the pre-slaughter phase, including crating and transportation (Adamczuk et al., 2014, Mohamed et al., 2014).

Stress, another affective state that may directly impact animal welfare, occurs when the animal perceives any physical or psychological situation as a threat to its homeostasis (Goldstein and Kopin, 2007). A stress response is followed by the activation of the hypothalamic-pituitary-adrenal (**HPA**) axis, where glucocorticoids and catecholamines are synthesized (Veissier and Boissy, 2007). Physiological changes derived directly from the activation of the HPA axis, such as changes in plasma corticosterone levels, can be measured to assess stress. However, those changes are short-term, and for that reason, the evaluation of chronic stress on poultry is often performed by the estimation of the H:L ratio (Gross and Siegel, 1983). A shift in leucocytes production is one of the many physiological responses that occurs in response to stress and aids in supporting the immune system (Davis and Maney, 2018). In our study, birds raised under blue light had a lower H:L ratio than birds raised under white light, suggesting a reduced stress level. This is in accordance with previous studies (Mohamed et al., 2017) and corresponds to the reduction in fear noted in this study.

A commonly held belief is that when poultry are raised under shorter wavelengths, such as blue light, behavioral expression may be impacted due to an effect of light wavelength on visual ability. This belief likely originates from considering humans' spectral sensitivity, where humans may not see as well when exposed to a short wavelength compared to white light. However, previous research demonstrated that when broilers are raised under blue light, they have improved spatial vision and minor changes in refraction index and eye health (Remonato Franco et al., 2022a). Therefore, poor vision is likely not the reason for any observed behavioral changes. In addition, differences in body weight or walking ability of the chickens could affect results of fear tests used in this study. However, light color had no significant effects on footpad dermatitis, gait scoring (Remonato Franco et al., University of Saskatchewan, Unpublished data), or body weight (Remonato Franco et al., 2022b).

Light can also affect non-visual brain responses that impact behavior and emotional states. Various wavelengths have specific capacities of activating non-retinal photoreceptors. According to Hartwig and van Veen (1979), light composed of long wavelengths is more capable of passing through the skull and reaching non-retinal photoreceptors. In contrast, light of short wavelength requires higher intensity to activate non-retinal photoreceptors in the hypothalamus. Wavelength may also impact melatonin levels and diurnal systems (Wright et al., 2004), which can be correlated to changes in behavior and affective states (Schwean-Lardner et al., 2013; Alzoki, 2019). In humans, light wavelength affects brain activity by changing how the brain processes different external stimuli and how fast the brain promotes behavioral adaptation to the environment (Vanderwalle et al., 2007, 2010). This specificity could lead to a novel explanation of why different wavelength treatments affect bird behavior if similar processes occur.

In conclusion, light color had a significant impact on broiler emotional states. For example, white light increased activity when compared to blue or green light, while blue light increased resting behavior. In addition, fear and stress levels were reduced for birds raised under blue light, which may indicate light color could serve as an important tool for managing broilers. However, the mechanisms of these behavioral changes are not fully understood, suggesting that more research is needed to understand the causes of changes in behavior, fear, and stress levels.
